# miRNA Expression Response of *Aedes aegypti* (Linnaeus 1762) (Diptera: Culicidae) to Imidacloprid Exposure

**DOI:** 10.3390/insects16050460

**Published:** 2025-04-27

**Authors:** Gerardo Trujillo-Rodríguez, Mariana Lizbeth Jiménez-Martínez, Elda Flores-Contreras, Everardo González Gonzalez, María de Lourdes Ramírez Ahuja, Idalia Garza Veloz, Adriana E. Flores Suarez, Fabian Correa Morales, Felipe Dzul Manzanilla, Iram P. Rodriguez Sanchez, Margarita L. Martínez Fierro

**Affiliations:** 1 Molecular Medicine Laboratory, Unidad Académica de Medicina Humana y C.S., Universidad Autónoma de Zacatecas, Zacatecas 98600, Zacatecas, Mexico; entogerry36@gmail.com (G.T.-R.); dnarnaprot@gmail.com (E.G.G.); idaliagv@uaz.edu.mx (I.G.V.); 2 Laboratorio de Fisiología Molecular y Estructural, Facultad de Ciencias Biológicas, Universidad Autónoma de Nuevo León, Av. Universidad S/N Ciudad Universitaria, San Nicolás de los Garza 66451, Nuevo León, Mexico; mariana.jimenez80@gmail.com (M.L.J.-M.); lulu.ahuja@hotmail.com (M.d.L.R.A.); 3 Departamento de Patología, Facultad de Medicina, Universidad Autónoma de Nuevo León, Francisco I. Madero y Dr. E. Aguirre Pequeño s/n, Mitras Centro, Monterrey 64460, Nuevo León, Mexico; elda.florescn@uanl.edu.mx; 4 Laboratorio de Entomología Medica, Facultad de Ciencias Biológicas, Universidad Autónoma de Nuevo León, Av. Universidad S/N Ciudad Universitaria, San Nicolás de los Garza 66451, Nuevo León, Mexico; adrflores@gmail.com; 5 Dirección del Programa de Enfermedades Transmitidas por Vectores del Centro Nacional de Programas Preventivos y Control de Enfermedades (CENAPRECE), Mexico City 01480, Mexico; fabiancorrea@msn.com (F.C.M.); felipe.dzul.m@gmail.com (F.D.M.)

**Keywords:** *Aedes aegypti*, miRNAs, imidacloprid, insecticide resistance, stress response, vector control

## Abstract

*Aedes aegypti* is a major vector of arboviruses, including dengue, Zika, chikungunya, yellow fever, and Mayaro. The extensive use of insecticides has led to resistance, complicating vector control efforts. MicroRNAs (miRNAs) are post-transcriptional regulators involved in detoxification and stress adaptation; however, their role in the insecticide response of *Ae. aegypti* remains unclear. This study analyzed miRNA expression in *Ae. aegypti* following imidacloprid exposure, comparing a field strain, Martinez de la Torre from Mexico (MT), and a susceptible reference strain, New Orleans (NO). Small RNA sequencing identified 96 miRNAs, with miR-1, miR-281-5p, miR-100, and miR-184 being consistently expressed across all conditions. In contrast, let-7, miR-124, and miR-13-3p were exclusively detected in wild mosquitoes exposed to imidacloprid, while miR-14 and miR-275-3p showed condition-specific expression. Distinct miRNA expression patterns were observed between the field and susceptible strains, with specific miRNAs showing condition-dependent expression. These findings suggest that certain miRNAs may serve as potential biomarkers for resistance monitoring, contributing to a better understanding of insecticide response mechanisms and informing novel vector control strategies.

## Simple Summary

Mosquitoes transmit serious diseases, including malaria, dengue, Zika, chikungunya, and yellow fever, among others, affecting hundreds of millions of people worldwide. Insecticides are widely used to control mosquito populations, but resistance development reduces their effectiveness over time. Understanding mosquitoes’ responses to insecticides at the molecular level can help to improve control strategies. This study examined microRNAs, small molecules that regulate gene activity and influence responses to environmental stress, including exposure to insecticides. We analyzed the following two *Aedes aegypti* (Linnaeus) populations: a laboratory strain susceptible to insecticides and a field population. After exposing these mosquitoes to imidacloprid (1 µg/mL), we assessed changes in microRNA expression. Our results identified microRNAs present in all mosquitoes and others only expressed in wild mosquitoes following insecticide exposure. These findings suggest that specific microRNAs may be associated with insecticide response and potentially linked to resistance mechanisms. Identifying molecular markers can help to develop improved mosquito control strategies, reduce insecticide resistance, and improve public health interventions. Understanding how mosquitoes adapt to insecticides is essential for designing new vector management approaches.

## 1. Introduction

*Ae. aegypti* is a primary vector of viral diseases of significant public health concern, including dengue, Zika, yellow fever, chikungunya, and Mayaro [1]. This species exhibits a high dispersal capacity, and its eggs are resistant to adverse conditions, facilitating their persistence in diverse environments [1]. Additionally, environmental factors such as climate change have contributed to its global expansion [2]. The use of insecticide-based interventions remains a key component of mosquito control strategies; however, the emergence of resistance represents a growing challenge [1].

MicroRNAs (miRNAs) are small (~22 nucleotides) non-coding RNA molecules that post-transcriptionally regulate gene expression in eukaryotes [3]. In insects, miRNAs are involved in key biological processes, including metamorphosis, blood digestion, and responses to environmental stressors, including insecticides [2]. Several miRNAs have been identified in *Ae. aegypti* with well-characterized functions. For instance, aae-miR-18905 is involved in blood digestion and metamorphosis [4], aae-miR-3096 regulates sexual dimorphism [5], and aae-miR-3757 is implicated in virus–host interaction [6].

Recent studies have highlighted the role of miRNAs in insecticide response and resistance mechanisms, showing that their differential expression can influence key detoxification genes, such as cytochrome P450s (CYPs), glutathione S-transferases (GSTs), and esterases [7]. Insecticide exposure can induce changes in miRNA expression which not only affect resistance development, but also serve as potential biomarkers for toxicological responses in both target and non-target organisms [7]. These findings suggest that miRNAs may play a critical role in regulating metabolic pathways and mitigating the toxic effects of insecticides.

In addition, miRNAs have been directly implicated in insecticide resistance and immune regulation in mosquitoes, positioning them as promising molecular tools for vector control. For instance, miR-285, miR-932, and miR-278-3p have been linked to pyrethroid resistance in *Culex pipiens pallens* (Linnaeus) through the modulation of genes such as CYP6N23, CpCPR5, and CYP6AG11 [8,9]. In *Anopheles sinensis* (Wiedemann), 39 differentially expressed miRNAs were identified in pyrethroid-resistant strains, associated with key pathways like membrane transport, signal transduction, and xenobiotic metabolism [10].

Beyond resistance, miRNAs also influence immune responses. miR-305, for example, suppresses immune gene expression in *Anopheles gambiae* (Giles), facilitating *Plasmodium falciparum* infection and gut microbiota proliferation [11]. Building on these insights, a novel biocontrol approach was developed using entomopathogenic fungi engineered to express mosquito-immunosuppressive miRNAs, enhancing their effectiveness even against resistant strains [12]. A comprehensive review also highlighted that miRNA expression in mosquitoes is highly specific to species, sex, life stage, and tissue, and is involved in fundamental processes such as development, metabolism, and host–pathogen interaction [13].

Insecticide resistance in mosquitoes is a multifactorial process involving the differential expression of detoxification genes, cellular stress responses, and metabolic adaptations. However, the specific role of miRNAs in this process remains largely unexplored. This study aims to characterize the miRNA profile of *Ae. aegypti* following exposure to imidacloprid, a neonicotinoid insecticide, to identify the key miRNAs associated with insecticide response. Using next-generation sequencing, we analyzed changes in miRNA expressions under different exposure conditions, providing insights into their potential roles in resistance mechanisms and stress adaptation. Understanding these interactions could contribute to the development of more effective and environmentally sustainable vector control strategies.

## 2. Materials and Methods

### 2.1. Biological Material

In this study, the following two *Ae. aegypti* strains were used: a wild population collected in Martínez de la Torre, Veracruz, Mexico (20.060556, −97.054167), in 2021 (MT), and the reference susceptible strain New Orleans (NO) (Table 1). The wild MT strain had not been previously tested for susceptibility to imidacloprid, therefore, its resistance status to this insecticide remains unknown. Both strains were maintained in the insectary of the Laboratorio de Fisiología Molecular y Estructural, Facultad de Ciencias Biológicas, Universidad Autónoma de Nuevo León, under controlled conditions (temperature of 28 ± 2 °C, 12:12 h light/dark photoperiod, and 70  ±  2% relative humidity). The field strain was selected to explore potential differences in molecular responses to insecticides when compared to a laboratory-maintained susceptible strain.

### 2.2. CDC Bottle Bioassay

Bioassays were conducted to obtain surviving mosquitoes after exposure to imidacloprid, using a sub-lethal concentration to provoke sufficient stress to generate a change in the mirnomic profile. A total of 250 mosquitoes from the F2 generation, aged from three to five days post-emergence and fed only with a 10% sucrose solution, were used in 250 mL bottles with four replicates per population (25 mosquitoes each) and a control group. IMIDACLOPRID 18380 STD 10 mg (ACCUSTANDARD, CTR scientific, Monterrey, Mexico) was dissolved in molecular-grade acetone to prepare a stock solution. Serial dilutions were used to determine a sub-lethal concentration that would not result in more than 50% mortality in the NO strain. The inner surfaces of the bottles were coated with 1 mL of imidacloprid solution at a concentration of 1 µg/mL. Control bottles were treated with acetone only. Mortality was recorded using the NO strain. The same concentration was then applied to the wild-type (MT) strain, although the LC_50_ for this population was not determined, as that was not the purpose of the experiment. The use of the same concentration allowed for a larger number of surviving wild mosquitoes, enabling the extraction of enough RNA. Immediately after one hour of imidacloprid exposure, the surviving mosquitoes were sacrificed at −20 °C for total RNA extraction. For each experimental condition, pools of 12–15 surviving mosquitoes were used. RNA was extracted using the TRIzol method, and samples were preserved dry in 1.5 mL GenTegra RNAssure Quick Protocol tubes (GenTegra LLC, Pleasanton, CA, USA) until next-generation sequencing.

### 2.3. RNA Extraction and Sequencing

miRNA extraction from the mosquito specimens followed the protocol described by Rodríguez-Sanchez et al. [2], using the miRNeasy Mini Kit (Qiagen, Germantown, MD, USA) according to the manufacturer’s instructions. The extracted total RNA was treated with RQ1 RNase-Free DNase (Promega, San Luis Obispo, CA, USA) to remove genomic DNA traces.

RNA purity and integrity were assessed using spectrophotometry (Thermo Fisher, Waltham, MA, USA) and agarose gel electrophoresis. RNA samples meeting quality and quantity requirements were sent to BGI Global Genomics Services (Yantian District, Shenzhen, China) for small RNA sequencing (<30 base pairs) using Illumina Solexa technology with paired-end adapter ligation.

### 2.4. miRNA Distribution

miRNA identification and expression analysis were conducted using miRDeep2 [8], with the *Ae. aegypti* genome version AaegL5 as the reference. A minimum read length of 18 nt was used, and only miRNAs with a miRDeep score of ≥4 were retained, following the criteria of Friedländer et al. [14] and our own filtering standards.

For miRNA identification, only sequences detected by miRDeep2 that matched previously reported *Ae. aegypti* miRNAs were considered. A Venn diagram was generated using the ggvenn package [15] in R [16] to illustrate the miRNAs’ distribution across experimental conditions (susceptible NO and field MT strains).

To visualize the miRNA expression patterns, a heatmap was constructed based on the top 10 most highly expressed miRNAs per sample. The heatmap was generated using the heatmaply [17] and RColorBrewer [18] packages in R [16], providing a comparative overview of miRNA expression across conditions.

## 3. Results

The LC50 was determined in CDC bottle bioassays for the NO strain against imidacloprid, which corresponded to a concentration of (1 µg/mL).

**Figure 1:**
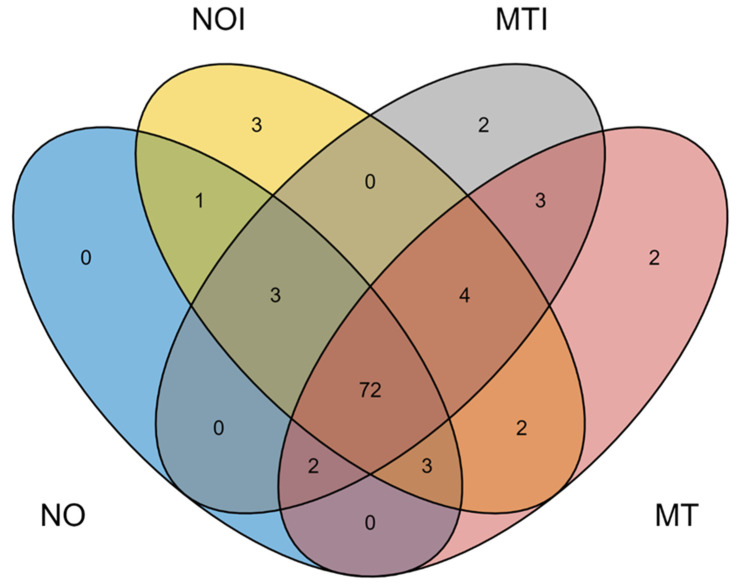
Venn diagram showing the shared and unique microRNAs across experimental conditions: NO (New Orleans strain without insecticide exposure), MT (Martínez de la Torre strain without insecticide exposure), NOI (New Orleans strain exposed to imidacloprid), and MTI (Martínez de la Torre strain exposed to imidacloprid). The numbers indicate the number of microRNAs found exclusively in each strain or shared among different groups. The central overlap (n = 72) represents the microRNAs common to all four approaches.

**Figure 2:**
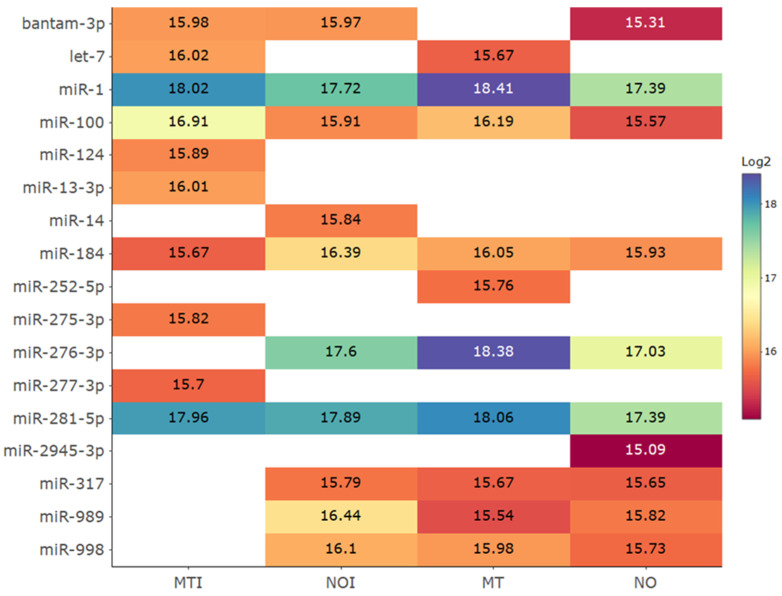
Heatmap of the top 10 expressed miRNAs in each condition: NO (New Orleans strain without insecticide exposure), MT (Martínez de la Torre strain without insecticide exposure), NOI (New Orleans strain exposed to imidacloprid), and MTI (Martínez de la Torre strain exposed to imidacloprid). Expression levels are shown in log2 scale for better visualization of differences.

Data analysis revealed multiple miRNAs that were either shared across conditions or uniquely expressed in specific conditions, suggesting potential regulatory roles in insecticide response and resistance. A total of 96 miRNAs were detected across all conditions, distributed into eleven different expression patterns. Among these, 72 miRNAs were shared across all conditions, including the susceptible NO strain and the field strain MT without insecticide exposure, as well as the same strains after imidacloprid exposure (NOI and MTI) (Figure 1). Additionally, four miRNAs were shared among three conditions (NOI, MT, and MTI), suggesting potential roles in insecticide exposure. Three miRNAs were exclusive to NOI (susceptible mosquitoes exposed to insecticide), possibly activating resistance-related genes. Another three miRNAs were shared between MT and MTI, potentially regulating genes linked to pre-existing resistance mechanisms. Furthermore, two miRNAs were exclusive to MT (wild-type mosquitoes in baseline conditions), suggesting a role in maintaining genetic resistance. Several unique miRNAs were identified in specific conditions, providing insights into their potential regulatory functions in stress responses and resistance. To refine these findings, the top 10 most highly expressed miRNAs per condition were analyzed (Figure 2). The most abundant miRNA was miR-1, detected in all conditions with log2 expression values ranging from 17.39 to 18.41. miR-281-5p also showed a high and consistent expression across groups, ranging from 17.39 to 18.06. Other consistently detected miRNAs included miR-100 (15.57–16.91) and miR-184 (15.67–16.39).

Some miRNAs were present in three of the four experimental groups, including miR-276-3p, miR-998, miR-317, miR-989, and bantam-3p, with log2 expression values varying between 15.31 and 18.38. In contrast, seven miRNAs appeared exclusive to a single condition. In MTI, these included miR-13-3p (16.01), miR-124 (15.89), miR-277-3p (15.70), and miR-275-5p (15.82). miR-14 was found only in NOI (15.84), miR-252-5p only in MT (15.76), and miR-2945-3p only in NO (15.09).

## 4. Discussion

Mosquito populations are constantly exposed to environmental stressors such as habitat modification, climate change, and the widespread use of insecticides for vector control [1]. To cope with these pressures, insects regulate their gene expression through various molecular mechanisms, among which miRNAs have emerged as key post-transcriptional regulators [2]. These small RNAs influence physiological processes such as development, reproduction, and responses to environmental stressors, including insecticide exposure [3].

In this study, 96 miRNAs were identified in *Ae. aegypti* under different experimental conditions, including a susceptible laboratory strain (NO) and a field strain (MT), both under normal conditions and following exposure to imidacloprid. Among these, miR-1, miR-281-5p, miR-100, and miR-184 were detected across all conditions, suggesting their role in mosquito physiology. In contrast, let-7, miR-124, and miR-13-3p, were exclusively expressed in field mosquitoes exposed to insecticide, indicating a potential link to stress adaptation or resistance mechanisms. Additionally, miR-14 and miR-275-3p were detected in specific conditions (approaches with imidacloprid exposure), suggesting condition-dependent regulatory roles.

### 4.1. miRNAs Associated with Metabolism and Cellular Regulation

Mosquitoes exposed to environmental stressors require tight regulatory control of their metabolic processes to maintain homeostasis. In this study, let-7 was exclusively detected in field mosquitoes (MT and MTI), aligning with previous reports associating let-7 with metabolic regulation [19]. This miRNA has been extensively studied across different organisms due to its role in development, metabolism, and even oncogenesis in mammals. Its absence in the susceptible strain (NO) suggests potential metabolic differences between laboratory-maintained and field mosquito populations.

In contrast, miR-1, which was present in all conditions, is a key regulator of muscle tissue function and thermal stress response [20]. Its consistently high expression levels across all groups may be linked to cold exposure during sample processing, a phenomenon previously described [21]. Another widely expressed miRNA, miR-281-5p, has been linked to ecdysone regulation and developmental processes in insects [21], further emphasizing the potential role of these miRNAs in maintaining physiological homeostasis.

### 4.2. miRNAs Linked to Insecticide Resistance and Response

Insecticide exposure induces molecular responses that mediate either resistance mechanisms or general stress responses. In this study, miR-13-3p was exclusively detected in field mosquitoes exposed to imidacloprid (MTI), reinforcing previous findings that have associated this miRNA with insecticide resistance in *Spodoptera frugiperda* [22]. Additionally, miR-276-3p, detected in three conditions (MTI, NOI, and NO), has been reported as a CYP6CX3 regulator in cyantraniliprole resistance [23], suggesting a similar role in imidacloprid response.

Among the widely detected miRNAs, miR-100 has been linked to the post-transcriptional regulation of CYP6CY3, a gene involved in insecticide metabolism [24]. Its presence across all conditions suggests that it may play a role in the general detoxification response in *Ae. aegypti*. Another relevant miRNA, miR-998, was detected in three experimental conditions, but was absent in MTI. This miRNA has been previously associated with resistance to *Bacillus thuringiensis* (Bti) toxins, where a higher expression correlates with lower resistance [25]. The absence of miR-998 in MTI, a field strain previously reported as resistant, suggests that its expression could be linked to susceptibility rather than resistance.

Previous studies have demonstrated that miRNAs regulate detoxification pathways by targeting cytochrome P450s, glutathione S-transferases (GSTs), and carboxylesterases [7]. The detection of miR-100 and miR-276-3p, both previously associated with detoxification, supports their potential roles in insecticide metabolism in *Ae. aegypti*.

Several miRNAs play key roles in regulating insecticide resistance and responses to environmental stress in insects by modulating genes involved in detoxification, development, and metabolism. The miRNA let-7, which, in our study, was detected only in the MT strain, both with and without exposure to imidacloprid, has been implicated in regulating genes such as CYP6CY3, which is associated with nicotine tolerance in *Myzus persicae* (Börner). A reduced expression of let-7 correlates with an increased expression of CYP6CY3, thereby enhancing resistance to plant toxins [24]. In combination with miR-100, let-7 also regulates wing morphogenesis in *Blattella germanica* (Linnaeus) and has been linked to increased longevity and metabolic alterations in *Drosophila melanogaster* (Maigen) [26,27].

Additionally, miR-13b-3p, which, in our study, was exclusively detected in the MT strain after exposure to imidacloprid (MTI), was identified as a regulator of insecticide tolerance in *Spodoptera frugiperda* (J. E. Smith), where its overexpression increased mortality following exposure to compounds such as cyantraniliprole and emamectin benzoate [22]. miR-14, associated in our data with the NO strain after exposure to imidacloprid (NOI), has been shown to regulate P450 genes such as CYP307a1, a key player in metabolic resistance in *Plutella xylostella* (Linnaeus) [28]. Another functionally relevant miRNA is miR-184, which was consistently expressed across all of our experimental conditions. Its overexpression was shown to facilitate infection by rice black-streaked dwarf virus in *Laodelphax striatellus* (Fallén) through the suppression of the immune-related gene *Ken* [29].

### 4.3. miRNAs Involved in Development and Reproduction

Reproductive processes in mosquitoes are tightly regulated, often in response to environmental factors. miR-14, which has been implicated in egg-laying regulation in *Apis mellifera* [30], was exclusively detected in the NOI condition (susceptible mosquitoes exposed to insecticide). Despite all females being in the same post-emergence stage and subjected to controlled dietary conditions, the presence of miR-14 in NOI suggests a potential role in oogenesis under insecticide stress.

Another miRNA associated with reproduction, miR-275-3p, was detected in MTI. Previous studies in *Nilaparvata lugens* (Hemiptera: Delphacidae) have linked this miRNA to reproductive regulation [31]. As this study focused on insecticide exposure, rather than reproductive processes, the detection of miR-275-3p in MTI suggests that it may also be involved in other physiological responses beyond reproduction. Additionally, miR-124, an miRNA crucial for neuromuscular and behavioral development in *Drosophila* (Diptera: Drosophilidae) [32], was exclusively detected in MTI, indicating a potential role in regulating stress responses specific to field mosquitoes exposed to insecticides.

### 4.4. miRNAs Associated with Stress Response and Physiological Plasticity

Insects respond to environmental stress through regulatory networks that control metabolism, immunity, and survival. miR-184, detected in all experimental conditions, has been reported to mediate interactions between baculoviruses and insect hosts [33]. Its consistent expression across groups suggests a potential role in general stress-related processes in *Ae. aegypti*.

Another key miRNA, miR-317, was overexpressed in three conditions (NO, NOI, and MT), but absent in MTI. In *Drosophila*, miR-317 regulates Toll pathway signaling, and its overexpression has been associated with reduced survival rates [34]. The absence of miR-317 in MTI may indicate an adaptive advantage in field populations, potentially contributing to insecticide tolerance. Similarly, miR-998, detected in NO, NOI, and MT, has been linked to low pupal eclosion rates and developmental malformations in *Aedes albopictus* [35]. Although this study did not analyze pupal stages, the high expression of miR-989 in these groups suggests a potential impact on adult fitness after insecticide exposure.

A particularly relevant miRNA, miR-277-3p, was exclusively detected in MTI. This miRNA has been associated with diapause regulation in *Culex pipiens* [36]. Its absence in other conditions suggests that diapause-related pathways may be suppressed in field mosquitoes exposed to insecticides.

Beyond vector populations, insecticide-induced miRNA expression changes have been reported in non-target organisms, including pollinators and aquatic species, raising concerns about broader ecological impacts [7]. Investigating miRNA-mediated responses across diverse taxa could enhance our understanding of insecticide toxicity beyond resistance mechanisms.

## 5. Conclusions

The characterization of miRNA expression profiles in *Ae. aegypti* under different insecticide exposure conditions provides valuable insights into their regulatory roles. Several miRNAs, including miR-1, miR-281-5p, and miR-100, were consistently expressed across all conditions, suggesting their involvement in fundamental physiological functions. In contrast, condition-specific miRNAs, such as miR-13-3p, miR-124, and miR-276-3p, were primarily detected in field mosquitoes exposed to imidacloprid, supporting their potential role in stress response and resistance mechanisms.

These findings contribute to a better understanding of the molecular basis of the insecticide response in *Ae. aegypti* and highlighting potential miRNA biomarkers for monitoring resistance. Future research should focus on the functional validation of these miRNAs and their specific targets, paving the way for novel strategies for mosquito control and resistance management.

## Figures and Tables

**Table 1:** Description of experimental groups and treatments.

Strain	Treatment	Abbreviation
New Orleans (susceptible)	Control (acetone only)	NO
New Orleans (susceptible)	Exposed to imidacloprid	NOI
Martínez de la Torre (wild)	Control (acetone only)	MT
Martínez de la Torre (wild)	Exposed to imidacloprid	MTI

## Data Availability

The datasets used and analyzed during the current study are available at: https://doi.org/10.5281/zenodo.14941652.

## Abbreviations

The following abbreviations are used in this manuscript:

NO	New Orleans strain
NOI	New Orleans strain exposed to imidacloprid
MT	Martinez de la Torre strain
MTI	Martinez de la Torre strain exposed to imidacloprid
